# Supplementary figures and images for: SARS-CoV-2 variant introduction following spring break travel and transmission mitigation strategies
Source: PLoS One. 2024 May 9;19(5):e0301225. doi: 10.1371/journal.pone.0301225 (PMC11081374; doi:10.1371/journal.pone.0301225)

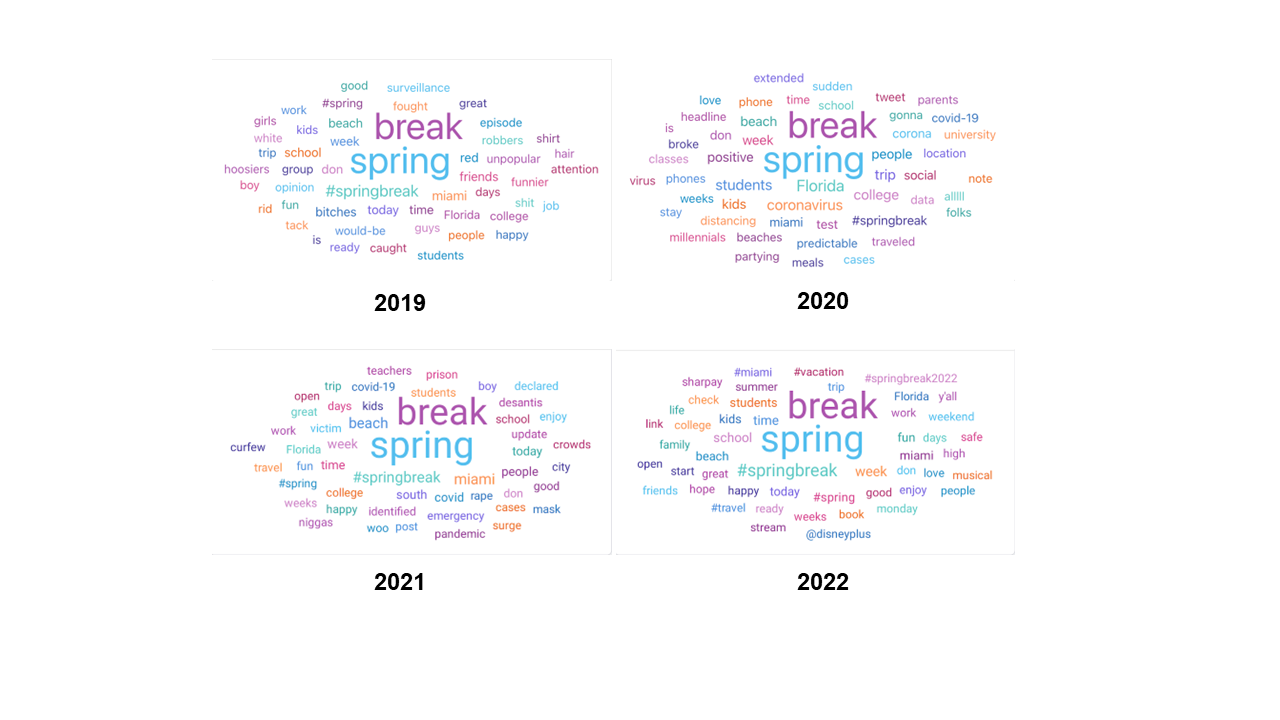

Supplement: S1 Fig — Four representations of the frequency of various words used during March and April of 2019, 2020, 2021, and 2022. Data was pulled from X (formerly Twitter) and Instagram. (TIF) [file pone.0301225.s001.tif]
